# Height of non‐Hispanic white adults with homeostatic iron regulator *HFE* genotypes p.C282Y/p.C282Y and wt/wt

**DOI:** 10.1002/mgg3.2321

**Published:** 2023-11-06

**Authors:** James C. Barton, J. Clayborn Barton, Ronald T. Acton

**Affiliations:** ^1^ Department of Medicine University of Alabama at Birmingham Birmingham Alabama USA; ^2^ Southern Iron Disorders Center Birmingham Alabama USA; ^3^ Department of Microbiology University of Alabama at Birmingham Birmingham Alabama USA

**Keywords:** age, hemochromatosis, iron, serum ferritin, transferrin saturation, weight

## Abstract

**Background:**

We sought to evaluate height in white adults with hemochromatosis.

**Methods:**

We analyzed the height of (1) post‐screening examination participants with *HFE* p.C282Y/p.C282Y (rs1800562) and wt/wt (absence of p.C282Y and p.H63D (rs1799945)) and (2) referred hemochromatosis probands with p.C282Y/p.C282Y.

**Results:**

There were 762 participants (270 p.C282Y/p.C282Y, 492 wt/wt; 343 men, 419 women) and 180 probands (104 men, 76 women). Median height of male participants with p.C282Y/p.C282Y or wt/wt was 177.8 cm. Median height of female participants was greater in those with p.C282Y/p.C282Y than wt/wt (165.1 cm vs 162.6 cm, respectively; *p* = 0.0298). Median height of p.C282Y/p.C282Y participants and probands was the same (men 177.8 cm; women 165.1 cm). Regressions on height of male and female participants revealed no associations with *HFE* genotype and inverse and positive associations with age and weight, respectively. Height of female participants was positively and inversely associated with transferrin saturation and serum ferritin, respectively. Regressions on height of male and female probands revealed positive associations with weight.

**Conclusions:**

The height of men with *HFE* p.C282Y/p.C282Y and wt/wt does not differ significantly. The height of female participants was greater in those with p.C282Y/p.C282Y than wt/wt. We found no independent association of *HFE* genotype with the height of men or women.

## Graphical abstract

We studied the height of adults with hemochromatosis genotype *HFE* p.C282Y/p.C282Y and without common *HFE* mutations (wt/wt). The median height of men with either genotype did not differ significantly, whereas the median height of women with *HFE* p.C282Y/p.C282Y was significantly greater than that of women with *HFE* wt/wt.

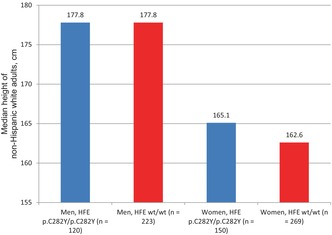

## 1 INTRODUCTION

Hemochromatosis in whites of western European descent is associated with homozygosity for p.C282Y (rs1800562), a common missense allele of the *HFE* gene (homeostatic iron regulator, chromosome 6p22.2; OMIM accession *613609; NM_000410.4(HFE):c.845G>A (p.Cys282Tyr)) in linkage disequilibrium with human leukocyte antigen (HLA) A*03 (Barton et al., 2015; Feder et al., 1996). HFE, a non‐classical class I major histocompatibility complex protein, is an upstream regulator of hepcidin, and thus of iron homeostasis (Rishi et al., 2015). Laboratory phenotypes of adults at diagnosis of *HFE* p.C282Y/p.C282Y often include elevated levels of transferrin saturation (TS) and serum ferritin (SF) (Edwards & Barton, 2019). Adults with p.C282Y/p.C282Y have increased risks of developing iron overload and resulting complications, including arthropathy, diabetes mellitus, hypogonadotropic hypogonadism, hepatic cirrhosis, and cardiomyopathy (Edwards & Barton, 2019). Severe iron overload occurs predominantly in men (Barton et al., 2018; Beaton et al., 2002; Edwards & Barton, 2019; Franchini, 2003). Non‐*HFE* heritable and environmental variables modify iron loading in adults with hemochromatosis and p.C282Y/p.C282Y (Barton et al., 2015; Edwards & Barton, 2019; Martin et al., 2020; Wood et al., 2008)

In a study of 176 Swiss adults with hemochromatosis (93% *HFE* p.C282Y/p.C282Y, 7% p.C282Y/p.H63D (rs1799945)) at University Hospital Zurich, height of 120 men (178.2 cm) was significantly greater than that of 458,322 men in an age‐ and sex‐matched Swiss reference population (173.9 cm) (Cippà & Krayenbuehl, 2013). Height of 56 women (167.1 cm) was also significantly greater than that of 10,260 women in a Swiss reference population (163.8 cm) (Cippà & Krayenbuehl, 2013).

Aims of this study were: (1) to evaluate associations of height of 762 non‐Hispanic white adults (270 *HFE* p.C282Y/p.C282Y, 492 *HFE* wt/wt (absence of p.C282Y and p.H63D)) with the variables sex, age, TS, SF, and weight determined in a post‐screening clinical examination of the North American primary care‐based Hemochromatosis and Iron Overload Screening (HEIRS) Study; and (2) to confirm associations of height of 180 referred non‐Hispanic white adult hemochromatosis probands with p.C282Y/p.C282Y with the same variables. We compare our observations with those of previous studies and discuss the associations of adult height with age, TS, SF, and weight.

## 2 MATERIALS AND METHODS

### 2.1 Ethical compliance: HEIRS Study

The HEIRS Study, funded by the National Heart, Lung, and Blood Institute/National Human Genome Research Institute, in accordance with principles of the Declaration of Helsinki, evaluated diverse aspects of hemochromatosis and iron overload in a primary care‐based sample of 101,168 adults enrolled during the interval 2001–2003 at four Field Centers in the United States and one in Canada (Adams et al., 2005). Local Institutional Review Boards of the HEIRS Study Coordinating Center, Central Laboratory, and each Field Center approved the Study protocol that is described in detail elsewhere (Adams et al., 2005; McLaren et al., 2003, 2008). Participants ≥25 y of age and able to give written informed consent were recruited from outpatient facilities associated with five Field Centers and gave written informed consent for initial screening and post‐screening clinical examination (Adams et al., 2005; McLaren et al., 2008).

### 2.2 Ethical compliance: Alabama hemochromatosis probands

This retrospective work was performed according to the principles of the Declaration of Helsinki. Performance of this study was approved by Western Institutional Review Board, Inc. (submission 2539985–44189619). Western Institutional Review Board, Inc. waived the need for obtaining informed consent from participants in this study under United States Department of Health and Human Services, Office for Human Research Participants, regulation 45 CFR 46.101(b)(4). Obtaining informed consent was not required and thus was not obtained because this study involved retrospective chart review and analyses of observations recorded in routine medical care. Data analyzed in this study were not anonymized before the investigators accessed them because data were compiled from proband charts in an Alabama tertiary hematology center, wherein JaCB diagnosed and treated all probands, consistent with Western Institutional Review Board, Inc. approval of this study. JaCB and JClB had access to information that could identify individual probands during or after data collection. All data in this report are displayed in a manner that maintains proband anonymity in both the present results and corresponding dataset.

### 2.3 Primary care‐based screening: HEIRS Study

The HEIRS Study recruited participants from a health maintenance organization, diagnostic blood collection centers, and public and private primary care offices in ambulatory clinics associated with five Field Centers (Adams et al., 2005). Ninety‐eight percent of self‐reported non‐Hispanic whites were recruited at Field Centers in Alabama, California, Ontario, and Oregon/Hawaii (Barton et al., 2005). Participation rates of non‐Hispanic white *HFE* p.C282Y homozygotes in primary care‐based screening were significantly greater than estimated by Hardy–Weinberg proportions at four of five Field Centers (Adams et al., 2005). Laboratory testing at screening included only TS and SF phenotyping and *HFE* p.C282Y (OMIM accession *613609; NM_000410.4(HFE):c.845G>A (p.Cys282Tyr)) and p.H63D allele‐specific genotyping (Adams et al., 2005).

HEIRS Study recruiters solicited participation of men and women equally. The greater proportion of women than men in the present study (55.0% vs 45.0%; *p* < 0.0001) is representative of the greater proportion of women than men who participated in HEIRS Study initial primary care‐based screening (62.9% vs 37.1%, respectively; *p* < 0.0001) (Adams et al., 2005). This is consistent with previous reports that women use more health care services than men (Bertakis et al., 2000). The prevalence of *HFE* p.C282Y/p.C282Y in non‐Hispanic white men and women who participated in the HEIRS Study did not differ significantly (Barton et al., 2005).

All participants reported their race/ancestry categories defined by the HEIRS Study and approved by the National Heart, Blood, and Lung Institute/National Human Genome Research Institute (Adams et al., 2005; McLaren et al., 2003). Of 299 *HFE* p.C282Y homozygotes detected, 94.0% reported non‐Hispanic white ancestry. Prevalences (95% confidence intervals) of p.C282Y homozygotes in race/ethnicity groups included: non‐Hispanic Whites 0.44 (0.42–0.47), Native Americans 0.11 (0.0061–0.20), Hispanics 0.027 (0.022–0.032), Blacks/African Americans 0.014 (0.012–0.017), Pacific Islanders 0.012 (0.0043–0.032), and Asians 0.000039 (0.000015, 0.00010) (Adams et al., 2005).

### 2.4 Post‐screening clinical examinations: HEIRS Study

Invitations to participate in post‐screening clinical examinations were extended to the following primary care‐based screening participants: (1) all with *HFE* p.C282Y/p.C282Y (regardless of screening TS and SF or previous diagnosis of hemochromatosis); (2) all whose screening TS and SF exceeded study thresholds (TS ≥ 55%, SF ≥ 300 μg/L (M); TS ≥ 45%, SF ≥ 200 μg/L (F), regardless of *HFE* genotype; and (3) those who had normal screening TS and SF and *HFE* wt/wt who were in the 25th–75th percentile of sex‐specific distributions and who were frequency‐matched for age (25–45 y, >45–65 y, and >65 y) and sex in a 1: 1 ratio with all participants with p.C282Y/p.C282Y identified at each of the five Field Centers (McLaren et al., 2003, 2008). A total of 2265 participants were invited to post‐screening clinical examinations. Of these, 1687 (74.5%) attended post‐screening clinical examinations (McLaren et al., 2008). Median interval between primary‐care‐based screening and post‐screening clinical examinations was 8 months (McLaren et al., 2003, 2008).

Post‐screening clinical examinations included the following: (1) questionnaires completed by participants that addressed medical history and medications; (2) focused physical examinations performed by HEIRS Study physicians; and (3) laboratory testing of blood specimens (McLaren et al., 2008).

The present cohort includes observations on all 762 non‐Hispanic whites who participated in post‐screening examinations and had either *HFE* p.C282Y/p.C282Y (*n* = 270) or wt/wt (*n* = 492), reported that they were not pregnant, and had complete data analyzed in this study. This cohort represents 94.7% (270/285) of non‐Hispanic white p.C282Y/p.C282Y clinical examination participants and 94.1% (492/523) of non‐Hispanic white wt/wt clinical examination participants (*p* = 0.7528).

### 2.5 Alabama referred hemochromatosis probands

We retrospectively compiled data on all consecutive self‐identified non‐Hispanic whites aged ≥18 y referred to an Alabama tertiary hematology center during the study interval 1 January 2007–30 October 2018 for evaluation and management of hemochromatosis who met the following criteria: (a) had *HFE* p.C282Y/p.C282Y, (b) had no known non‐hemochromatosis iron disorder, (c) underwent measurement of IgG subclasses at diagnosis, (d) underwent HLA‐A and ‐B typing, (e) achieved iron depletion with therapeutic phlebotomy, as appropriate, and (f) were the first in their respective families to be diagnosed to have hemochromatosis (probands).

Medical histories were taken from probands and records of referring physicians. Referring physicians diagnosed and treated probands with diabetes. Physicians in the present hematology center evaluated probands for cirrhosis, as appropriate. All probands underwent medication review, physical examination, laboratory testing, imaging procedures, and evaluation of liver and other conditions, as indicated, before therapeutic phlebotomy was initiated. Details of this cohort are reported elsewhere (Barton et al., 2022).

### 2.6 Laboratory methods: HEIRS Study

A morning blood sample was obtained from each post‐screening clinical examination participant after an overnight fast of ≥8 h to minimize diurnal variation of TS and SF (Dale et al., 2002). Testing measurements included serum TS and SF (Hitachi 911 Analyzer, Roche Applied Science, Indianapolis, IN, USA) as described in detail elsewhere (McLaren et al., 2008). *HFE* p.C282Y and p.H63D genotypes were confirmed using spots of whole blood and a modification of the Invader assay (Third Wave Technologies, Madison, WI, USA) that increases the allele‐specific fluorescent signal by including 12 cycles of locus‐specific polymerase chain reaction before the cleavase reaction (Adams et al., 2005).

### 2.7 Laboratory methods: Alabama hemochromatosis probands

TS and SF were measured using morning specimens obtained without regard to fasting and standard clinical laboratory methods (Laboratory Corporation of America, Burlington, NC, USA). *HFE* genotypes were determined as described in detail elsewhere (Barton et al., 1997).

### 2.8 Height and weight measures

Height and weight were measured with stadiometers and balance‐beam scales, respectively. Height and weight were recorded to the nearest inch and the nearest pound, respectively. Herein, we display height as centimeters (1.0 inch = 2.54 cm) and weight as kilograms (1.0 pound = 0.454 kg).

### 2.9 Literature searches

We performed computerized and manual searches to identify publications that contain observations regarding associations of the terms height, weight, children, and adults with terms hemochromatosis, *HFE* p.C282Y, p.C282Y/p.C282Y, rs1800562, SF, and TS.

### 2.10 Statistics

The datasets for analyses consisted of post‐screening examination observations in 762 participants (270 *HFE* p.C282Y/p.C282Y, 492 wt/wt) and observations at diagnosis of 180 referred Alabama hemochromatosis probands with p.C282Y/p.C282Y. There were no missing data. Age, TS, and SF measures are displayed to the nearest integer. Height and weight measures are displayed to the nearest tenth. Descriptive data are displayed as enumerations, percentages, means (±1 standard deviation (SD)), or medians (range). Analyses of continuous data using Kolmogorov–Smirnov and Shapiro–Wilk tests revealed that the distribution of age values did not differ significantly from normal and thus these values are displayed as mean ± 1 SD and compared using Student's *t* test for unpaired data (two‐tailed). Continuous variables that were not normally distributed (TS, SF, height, and weight) are displayed as median (range) and were compared initially using Mann–Whitney *U* test (two‐tailed), a measure of mean ranks (Hart, 2001). We also compared height data in post‐screening examination participants after *z*‐transformation using Student's *t* test for unpaired data (two‐tailed). Percentages were compared using Fisher's exact test (two‐tailed).

We prepared smoothed frequency distribution of height measures intervals of men and women with *HFE* p.C282Y/p.C282Y homozygosity and wt/wt who participated in a post‐screening clinical examination (95% CI of percentages (with continuity corrections)).

Height of men is greater than that of women from the same population (WorldData.info, 2023). TS and SF values of men with *HFE* p.C282Y/p.C282Y in the HEIRS Study post‐screening clinical examinations (McLaren et al., 2008) and referred hemochromatosis probands with p.C282Y/p.C282Y diagnosed in clinical practice (Edwards & Barton, 2019) are significantly higher than those of women from the same cohorts. Thus, we performed separate backward stepwise regressions on height of men and women using these independent variables: *HFE* genotype, age, TS, SF, and weight, as appropriate. Elimination of the last independent variable with *p* ≥ 0.05 was the stopping rule for regressions.

We defined *p* < 0.05 to be significant. We did not use a Bonferroni “correction” for univariate comparisons because we did not wish to reject “true positive” results (Armstrong, 2014). We used Excel® 2000 (Microsoft Corp., Redmond, WA, USA), GB‐Stat® (v. 10.0, Dynamic Microsystems, Inc., Silver Spring, MD, USA), and GraphPad Prism 8® (2018; GraphPad Software, San Diego, CA, USA).

## 3 RESULTS

### 3.1 General characteristics of post‐screening clinical examination participants

There were 270 participants with *HFE* p.C282Y/p.C282Y and 492 participants with *HFE* wt/wt. There were 343 men (Table 1) and 419 women (Table 2). Mean age of all participants was 55 ± 14 y. Mean ages of men and women p.C282Y/p.C282Y were significantly lower than those of men and women with wt/wt, respectively (Tables 1 and 2). Median TS and median SF were significantly higher in both men and women with p.C282Y/p.C282Y than in men and women with wt/wt, respectively (Tables 1 and 2).

**TABLE 1:** Characteristics of men in HEIRS Study post‐screening clinical examinations.^a^

Characteristic	*HFE* p.C282Y/p.C282Y (*n* = 120)	*HFE* wt/wt (*n* = 223)	*p* value
Mean age, y (standard deviation)	54 ± 14	57 ± 14	0.0171
Median transferrin saturation, % (range)	79 (7, 100)	35 (9, 97)	<0.00001
Median serum ferritin, μg/L (range)	555 (8, 5300)	195 (8, 3770)	<0.00001
Median height, cm (range)	177.8 (160.0, 200.7)	177.8 (160.0, 205.7)	0.0424^b^
Median weight, kg (range)	87.1 (54.3, 135.1)	86.7 (45.4, 165.7)	0.2684

^a^ HEIRS Study, Hemochromatosis and Iron Overload Screening Study; wt/wt, absence of *HFE* p.C282Y (rs1800562; (OMIM accession *613609; NM_000410.4(HFE):c.845G>A (p.Cys282Tyr))) and p.H63D (rs1799945).

^b^ Z‐transformed height measures of men with *HFE* p.C282Y/p.C282Y (mean 0.1195 ± 0.9871) and wt/wt (−0.0643 ± 1.0032) did not differ significantly (Student's *t*‐test (two‐tailed) *p* = 0.1033), confirming that height of men with p.C282Y/p/C282Y does not differ significantly from that of men with wt/wt.

**TABLE 2:** Characteristics of women in HEIRS Study post‐screening clinical examinations.^a^

Characteristic	*HFE* p.C282Y/p.C282Y (*n* = 150)	*HFE* wt/wt (*n* = 269)	*p* value
Mean age, y (standard deviation)	52 ± 13	56 ± 14	0.0017
Median transferrin saturation, % (range)	61 (6, 100)	29 (5, 96)	<0.00001
Median serum ferritin, μg/L (range)	221 (8, 5060)	71 (8, 2405)	<0.00001
Median height, cm (range)	165.1 (144.8, 180.3)	162.6 (137.2, 182.9)	0.0298^b^
Median weight, kg (range)	74.5 (44.9, 138.5)	71.7 (43.1, 135.3)	0.3732

^a^ HEIRS Study, Hemochromatosis and Iron Overload Screening Study; wt/wt, absence of *HFE* p.C282Y (rs1800562; (OMIM accession *613609; NM_000410.4(HFE):c.845G>A (p.Cys282Tyr)) and p.H63D (rs1799945).

^b^ Z‐transformed height measures in women with p.C282Y/p.C282Y (mean 0.1432 ± 1.0712) and wt/wt (−0.0798 ± 0.9508) also differed significantly (Student's *t*‐test (two‐tailed) *p* = 0.0346), confirming that height of women with p.C282Y/p/C282Y is greater than that of women with wt/wt.

In men with *HFE* p.C282Y/p.C282Y, 72.5% (87) had TS < 55% and 30.8% (37) had SF < 300 μg/L. In men with wt/wt, 11.7% (26) had TS ≥ 55% and 30.9% (69) had SF ≥ 300 μg/L. In women with p.C282Y/p.C282Y, 24.0% (36) had TS < 45% and 40.7% (73) had SF < 200 μg/L. In women with wt/wt, 10.0% (27) had TS ≥ 45% and 16.7% (45) had SF ≥ 200 μg/L.

### 3.2 Height and weight of post‐screening clinical examination participants

Median height of men with *HFE* p.C282Y/p.C282Y and wt/wt was the same (Table 1), although the difference of mean ranks of height measures of men with p.C282Y/p.C282Y and wt/wt was significant in a Mann–Whitney *U* test (*p* = 0.0424). Z‐transformed height measures of men with p.C282Y/p.C282Y (mean 0.1195 ± 0.9871) and wt/wt (−0.0643 ± 1.0032) did not differ significantly (Student's *t*‐test (two‐tailed) *p* = 0.1033), confirming that height of men with p.C282Y/p/C282Y does not differ significantly from that of men with wt/wt. Frequency distributions of height measures intervals in men with p.C282Y/p.C282Y and wt/wt are displayed in (Barton et al., 2023). Median weight of men with p.C282Y/p.C282Y and wt/wt did not differ significantly (Table 1).

Median height of women with *HFE* p.C282Y/p.C282Y was greater by 2.5 cm than that of women with wt/wt (Table 2), and the difference between mean ranks of height measures of women with p.C282Y/p.C282Y and wt/wt in a Mann–Whitney *U* test was significant (*p* = 0.0298). Z‐transformed height measures in women with p.C282Y/p.C282Y (mean 0.1432 ± 1.0712) and wt/wt (−0.0798 ± 0.9508) also differed significantly (Student's *t*‐test (two‐tailed) *p* = 0.0346), confirming that height of women with p.C282Y/p/C282Y is greater than that of women with wt/wt. Frequency distributions of height measures intervals in women with p.C282Y/p.C282Y and wt/wt are displayed in (Barton et al., 2023). Median weight of women with p.C282Y/p.C282Y and wt/wt did not differ significantly (Table 2).

### 3.3 Regressions on height of men and women in a post‐screening clinical examination

We performed backward stepwise regression on height of men using the independent variables *HFE* genotype (dichotomous), age, TS, SF, and weight. *HFE* genotype and SF were not significantly associated with height, after correction for other variables. Regression detected one inverse association: age (*p* = 0.0001). Regression detected one positive association: weight (*p* < 0.0001). This regression (ANOVA *p* < 0.0001) explained 28.4% of the variance of height of men in a post‐screening clinical examination.

We performed backward stepwise regression on height of women using the independent variables *HFE* genotype (dichotomous), age, TS, SF, and weight. *HFE* genotype was not significantly associated with height, after correction for other variables. Regression detected two inverse associations: age (*p* = 0.0082) and SF (*p* = 0.0120). Regression also detected two positive associations: TS (*p* = 0.0017), and weight (*p* < 0.0001). This regression (ANOVA *p* < 0.0001) explained 10.6% of the variance of height of women in a post‐screening clinical examination.

### 3.4 General characteristics of referred hemochromatosis probands

There were 104 men (57.8%) and 76 women (42.2%). All had *HFE* p.C282Y/p.C282Y. Mean age (SD) at diagnosis of hemochromatosis was 49 ± 13 y. Mean TS was 79 ± 17%. Median SF was 715 μg/L (28, 6103).

### 3.5 Comparisons of probands and post‐screening participants with *HFE* p.C282Y/p.C282Y

Median TS and median SF were significantly higher in referred male probands than male post‐screening clinical examination participants (Table 3), consistent with a previous report (Bulaj et al., 2000). Median TS and median SF were significantly higher in referred female probands than female post‐screening clinical examination participants (Table 4), consistent with a previous report (Bulaj et al., 2000). Differences between median TS of referred probands and post‐screening clinical examination participants may be due in part to differences in pre‐test fasting (Adams et al., 2007; Nguyen et al., 2017).

**TABLE 3:** Characteristics of men with *HFE* p.C282Y/p.C282Y.^a^

Characteristic	Alabama referred hemochromatosis probands (*n* = 104)	HEIRS Study^b^ post‐screening examination participants (*n* = 120)	*p* value
Mean age, y (standard deviation)	47 ± 14	54 ± 14	0.0004
Median transferrin saturation, % (range)	86 (43, 100)	79 (7, 100)	0.0105
Median serum ferritin, μg/L (range)	957 (28, 6103)	555 (8, 5300)	<0.0001
Median height, cm (range)	177.8 (149.9, 190.5)	177.8 (160.0, 200.7)	0.3592
Median weight, kg (range)	88.0 (54.3, 204.3)	87.1 (54.3, 135.1)	0.9840

^a^ *HFE* p.C282Y (rs1800562; (OMIM accession *613609; NM_000410.4(HFE):c.845G>A (p.Cys282Tyr)).

^b^ HEIRS Study, Hemochromatosis and Iron Overload Screening Study.

**TABLE 4:** Characteristics of women with *HFE* p.C282Y/p.C282Y.^a^

Characteristic	Alabama referred hemochromatosis probands (*n* = 76)	HEIRS Study^b^ post‐screening examination participants (*n* = 150)	*p* value
Mean age, y (standard deviation)	50 ± 13	52 ± 13	0.7566
Median transferrin saturation, % (range)	81 (39, 100)	61 (6, 100)	0.0002
Median serum ferritin, μg/L (range)	506 (32, 3427)	221 (8, 5060)	<0.0001
Median height, cm (range)	165.1 (152.4, 177.8)	165.1 (144.8, 180.3)	0.1967
Median weight, kg (range)	68.3 (46.7, 147.6)	74.5 (44.9, 138.5)	0.0324

^a^ *HFE* p.C282Y (rs1800562; (OMIM accession *613609; NM_000410.4(HFE):c.845G>A (p.Cys282Tyr)).

^b^ HEIRS Study, Hemochromatosis and Iron Overload Screening Study.

In men, median height of Alabama referred probands with *HFE* p.C282Y/p.C282Y and HEIRS Study post‐screening examination participants with p.C282Y/p.C282Y was the same (177.8 cm) (Table 3). Median weight did not differ significantly between these cohorts of men (Table 3).

In women, median height of Alabama referred probands with *HFE* p.C282Y/p.C282Y and HEIRS Study post‐screening examination participants with p.C282Y/p.C282Y was the same (165.1 cm) (Table 4). Median weight of Alabama referred probands was significantly lower than that of women who participated in post‐screening examinations (Table 4).

### 3.6 Regressions on height of referred hemochromatosis probands

In men, backward stepwise regression on height using the independent variables age, TS, SF, and weight detected one positive association: weight (*p* < 0.0001). This regression (ANOVA *p* < 0.0001) explained 15.7% of the variance of height of male Alabama referred probands.

In women, backward stepwise regression on height using the independent variables age, TS, SF, and weight detected one positive association: weight (*p* < 0.0032). This regression (ANOVA *p* < 0.0032) explained 11.1% of the variance of height of female Alabama referred probands.

## 4 DISCUSSION

Novel aspects of this report are analyses of height, TS, SF, and weight measures of (1) self‐identified non‐Hispanic white men and women with *HFE* genotypes p.C282Y/p.C282Y or wt/wt who participated in the North American primary care‐based HEIRS Study and (2) a cohort of referred Alabama non‐Hispanic white hemochromatosis probands with p.C282Y/p.C282Y. Median height of women with p.C282Y/p.C282Y (HEIRS Study participants and Alabama referred probands) was 165.1 cm. This height is significantly greater than median height of women with wt/wt (162.6 cm), greater than mean height of 2056 non‐Hispanic white women ages ≥20 y who participated in the United States National Health and Nutrition Examination Survey (NHANES) 1999–2002 (163.0 cm) (Ogden et al., 2004), and greater than height of women in North America (163 cm) (WorldData.info, 2023). Median height of the present men with either p.C282Y/p.C282Y (HEIRS Study participants and Alabama referred probands) or wt/wt (HEIRS Study participants) was 177.8 cm, greater than mean height of 2149 non‐Hispanic white men ages ≥20 y who participated in the NHANES 1999–2002 (177.2 cm) (Ogden et al., 2004), and greater than mean height of men in North America (177 cm) (WorldData.info, 2023).

Height of the present women with *HFE* p.C282Y/p.C282Y was significantly greater than height of women with wt/wt or height of women in other cohorts unselected for *HFE* genotypes, although we did not observe corresponding differences in height of men with and without p.C282Y/p.C282Y. In a study at University Hospital Zurich, height measures of men and women with hemochromatosis (93% p.C282Y/p.C282Y) were significantly greater than those of men and women in a Swiss reference population not evaluated for p.C282Y or p.H63D genotypes or TS or SF phenotypes (Cippà & Krayenbuehl, 2013). Differences between the present results and those of the study of Swiss adults may be due to genetic or non‐heritable factors.

Most variation in height is genetically controlled (Lango et al., 2010; Lettre, 2009; Locke et al., 2015). Frequencies of alleles associated with increased height, both at known loci and genome‐wide, are significantly greater in northern than southern Europeans (Turchin et al., 2012). The prevalence of *HFE* p.C282Y is also greater in northern than southern Europeans (Merryweather‐Clarke et al., 2000). It was conjectured that p.C282Y/p.C282Y increases height (Cippà & Krayenbuehl, 2013), although the present regression analyses revealed that height of men and women who participated in the HEIRS Study post‐screening clinical examinations is not significantly associated with p.C282Y/p.C282Y or wt/wt, after adjustment for other variables. We found no other report that rs1800562 (p.C282Y) is associated with height of humans (National Human Genome Research Institute and European Bioinformatics Institute, 2023). Lengths of mice homozygous for a p.C282Y knockin (*Hfe* ^*Y/Y*^) and control mice (*Hfe* ^*+/+*^) were not reported (Ajioka et al., 2002; Levy et al., 1999).

No linkage disequilibrium of *HFE* p.C282Y with height‐associated genes in all available population panels of the ‘International HapMap Project’ 5 and the ‘1000 Genomes Project’ 6 datasets using the SNAP search tool (http://www.broadinstitute.org/mpg/snap) was discovered by other investigators (Cippà & Krayenbuehl, 2013). These observations suggest that height of adults is not positively associated with a locus in linkage disequilibrium with p.C282Y.

No SNPs associated with hemochromatosis penetrance (Milet et al., 2007) that were also height‐related SNPs were reported (Cippà & Krayenbuehl, 2013). This observation suggests that height is not positively associated with a locus on a chromosome other than 6p that influences TS and SF in adults with *HFE* p.C282Y/p.C282Y.

Height of the present women who participated in the HEIRS Study post‐screening clinical examinations was significantly associated with TS, after adjustment for other variables. In contrast, height of the present Alabama referred probands with *HFE* p.C282Y/p.C282Y was not significantly associated with TS, after adjustment for other variables. In 7548 men aged ≥20 y in the United States, 1.7% (95% confidence interval (CI) 1.1–2.4) had TS >55%, although only 15.5% (95% CI: 9.0–22.1) of those also had elevated SF (>400 μg/L) (Looker & Johnson, 1998). In 8291 women aged ≥20 y in the United States, 4.7% (95% CI: 3.8–5.7) had TS >45%, although only 13.3% (95% CI: 5.6–21.1) of those also had elevated SF (>200 μg/L ages 20–49 y, >300 μg/L ages ≥50 y) (Looker & Johnson, 1998). Thus, elevated TS is common in adults in the United States, although most adults with elevated TS do not have iron overload.

Iron, an essential element for linear growth in children and adolescents (Soliman et al., 2009), is transported and delivered to cells via their surface transferrin receptors (Anderson & Frazer, 2017; Graham et al., 2012). It was conjectured that “patients with *HFE* hemochromatosis may benefit in their first two decades from constantly enhanced iron absorption, providing a steadily sufficient supply of iron during physical development” (Cippà & Krayenbuehl, 2013). TS in healthy subjects unselected for hemochromatosis or *HFE* genotypes is significantly lower in children than adults (Koerper & Dallman, 1977; Milman & Cohn, 1984). TS in iron‐replete children increases gradually with age (Milman & Cohn, 1984). In a male diagnosed to have p.C282Y/p.C282Y at age 10 y, TS remained elevated for 8 y thereafter in the absence of treatment (Porto et al., 2019), although we found no other longitudinal observations of TS in children with p.C282Y/p.C282Y. A systematic review of 25 randomized controlled trials that evaluated the effect of dietary iron supplementation on height and weight of children did not document a significant positive effect (Sachdev et al., 2006). Together, it is plausible that linear growth is greater in children whose TS is higher than normal, although this is unproven.

Height of the present women who participated in HEIRS Study post‐screening clinical examinations was inversely associated with SF, after adjustment for other variables, although the strength of this association was not great. We observed no significant association of height with SF in Alabama referred female probands or in men. Height of Swiss adults with hemochromatosis was not associated with SF (Cippà & Krayenbuehl, 2013). SF levels are directly related to the magnitude of bone marrow (Anupama et al., 2017; Rocha et al., 2009), liver (Olthof et al., 2007), and phlebotomy‐mobilizable (Beutler et al., 2002; Walters et al., 1973) iron stores. Elevated SF is usually due to acute or chronic inflammation, chronic alcohol consumption, liver disease, renal failure, metabolic syndrome, or malignancy, not iron overload (Koperdanova & Cullis, 2015).

Height and age were inversely associated in the present men and women who participated in HEIRS Study post‐screening clinical examinations, after adjustment for other variables. We did not observe a significant association of height with age in Alabama referred probands, although mean age of Alabama referred men with *HFE* p.C282Y/p.C282Y was significantly less than that of HEIRS Study men with p.C282Y/p.C282Y. In adults unselected for iron phenotypes or *HFE* genotypes, there was an inverse association of height with age (Cline et al., 1989).

Height and weight were positively associated in the present men and women who participated in HEIRS Study post‐screening clinical examinations and in Alabama referred probands with *HFE* p.C282Y/p.C282Y, after adjustment for other variables. This is consistent with observations in adults unselected for iron phenotypes or *HFE* genotypes, although the relationship of height and weight differs significantly between men and women (Diverse Populations Collaborative Group, 2005). Height of Swiss adults with hemochromatosis was not associated with body mass index (Cippà & Krayenbuehl, 2013).

Height of modern Europeans and derivative populations is greater than that of most other human populations (Scheffler & Hermanussen, 2022; WorldData.info, 2023). This difference is attributed to the inheritance of more height‐associated alleles or SNPs (especially in Northern Europeans and their descendants) (Turchin et al., 2012), to more advantageous socioeconomic conditions, to more favorable maternal and child nutrition, and to lower population burdens of illness in Europeans than other populations (Scheffler & Hermanussen, 2022). Consistent with these observations, the present regressions reveal that 71.6% and 89.4% of the variance of height of men and women, respectively, who participated in post‐screening clinical examinations and 84.3% and 88.9% of height variance of referred men and women, respectively, is explained by variables other than *HFE* genotypes, age, TS, SF, and weight. Adult height was also associated with increased odds ratios of atrial fibrillation, venous thromboembolism, intervertebral disc disorder, hip fracture, vasculitis, cancer overall, and breast cancer in a study of 417,434 white adults who participated in the United Kingdom Biobank (Lai et al., 2018).

A strength of the present study is the availability of (1) data from a large primary care‐based screening study in which post‐screening examination participants were selected for *HFE* genotypes, age, sex, TS, and SF and in whom *HFE* genotypes were confirmed and post‐screening TS and SF were measured, and (2) data for confirmation from a large cohort of Alabama‐referred hemochromatosis probands with *HFE* p.C282Y/p.C282Y. Investigating socioeconomic conditions, maternal and child nutrition, and the population burden of illness germane to the present subjects when they were children was not possible. Estimating odds ratios of other conditions positively associated with adult height and identifying alleles associated with height was beyond the scope of the HEIRS Study and the present referral practice.

## 5 CONCLUSIONS

We conclude that the height of men with *HFE* p.C282Y/p.C282Y and wt/wt does not differ significantly. The height of female participants was greater in those with p.C282Y/p.C282Y than wt/wt. We found no independent association of *HFE* genotype with the height of men or women.

## AUTHOR CONTRIBUTIONS

*Conceptualization; data curation; formal analysis; investigation; methodology; validation; visualization; writing – original draft preparation; writing – review & editing*: James C. Barton and J. Clayborn Barton. *Investigation; methodology; validation; visualization; writing – original draft preparation; writing – review & editing*: Ronald T. Acton.

## FUNDING INFORMATION

The authors received no specific funding for this particular work.

## CONFLICT OF INTEREST STATEMENT

None of the authors has a conflict of interest to report.

## ETHICS APPROVAL STATEMENT

Please see **MATERIALS AND METHODS**, first two paragraphs.

## PATIENT CONSENT STATEMENT

Please see **MATERIALS AND METHODS**, first two paragraphs.

## PERMISSION TO REPRODUCE MATERIAL FROM OTHER SOURCES

Not applicable.

## CLINICAL TRIAL REGISTRATION

Not applicable.

## Data Availability

HEIRS Study post‐screening clinical examination participants: The National Heart, Lung, and Blood Institute does not permit investigators to submit data directly to journal or related repositories or other sources. Parties interested in obtaining data analyzed in the present study are referred to the Biologic Specimen and Data Repository Information Coordinating Center (BioLINCC) (https://biolincc.nhlbi.nih.gov/studies/heirs/). Referred Alabama hemochromatosis probands with *HFE* p.C282Y/p.C282Y: A dataset and supplemental figures that correspond to the present analyses are available at Barton et al. (2023).
